# Meal and Sleep Timing before and during the COVID-19 Pandemic: A Cross-Sectional Anonymous Survey Study from Sweden

**DOI:** 10.3390/clockssleep3020015

**Published:** 2021-04-22

**Authors:** Christian Benedict, Luiz Eduardo Mateus Brandão, Ilona Merikanto, Markku Partinen, Bjørn Bjorvatn, Jonathan Cedernaes

**Affiliations:** 1 Department of Neuroscience, Sleep Science (BMC), Uppsala University, 752 36 Uppsala, Sweden; 2 Department of Medical Sciences, Uppsala University, 752 36 Uppsala, Sweden; luiz-eduardo.mateus-brandao@medsci.uu.se; 3 Department of Public Health Solutions, Finnish Institute for Health and Welfare, 00271 Helsinki, Finland; ilona.merikanto@helsinki.fi; 4 Department of Psychology and Logopedics, Faculty of Medicine, University of Helsinki, 00100 Helsinki, Finland; 5 Orton Orthopaedics Hospital, 00280 Helsinki, Finland; 6 Helsinki Sleep Clinic, Vitalmed Research Center, 00420 Helsinki, Finland; markpart@icloud.com; 7 Department of Neurosciences, Clinicum, University of Helsinki, 00100 Helsinki, Finland; 8 Department of Global Public Health and Primary Care, University of Bergen, 5009 Bergen, Norway; Bjorn.Bjorvatn@uib.no; 9 Norwegian Competence Center for Sleep Disorders, Haukeland University Hospital, 5021 Bergen, Norway; 10 Department of Medicine, Division of Endocrinology, Metabolism, and Molecular Medicine, Feinberg School of Medicine, Northwestern University, Chicago, IL 60611, USA

**Keywords:** COVID-19 pandemic, anonymous survey, meal timing, sleep timing, Sweden

## Abstract

The COVID-19 pandemic and related restrictions, such as stay-at-home-orders, have significantly altered daily routines and lifestyles. Given their importance for metabolic health, we herein compared sleep and meal timing parameters during vs. before the COVID-19 pandemic based on subjective recall, in an anonymous Swedish survey. Among 191 adults (mean age: 47 years; 77.5% females), we show that social jetlag, i.e., the mismatch in sleep midpoint between work and free days, was reduced by about 17 min during the pandemic compared with the pre-pandemic state (*p* < 0.001). Concomitantly, respondents’ sleep midpoint was shifted toward morning hours during workdays (*p* < 0.001). A later daily eating midpoint accompanied the shift in sleep timing (*p* = 0.001). This effect was mainly driven by a later scheduled first meal (*p* < 0.001). No difference in the timing of the day’s last meal was found (*p* = 0.814). Although our survey was limited in terms of sample size and by being cross-sectional, our results suggest that the delay in sleep timing due to the COVID-19 pandemic was accompanied by a corresponding shift in the timing of early but not late meals.

## 1. Introduction

The novel coronavirus SARS-CoV-2, the causative agent of Coronavirus disease 2019 (COVID-19), was first identified at the end of 2019 in China and has since spread across the world [1]. Due to its lethality, which log-linearly rises with age [2], many countries worldwide have taken action to slow the spread of the virus, including stay-at-home, work-from-home, and social distancing orders. These social restrictions have come at a cost, including reports of reduced well-being, increasing mental health issues, and lower physical activity levels [3,4]. On the other hand, working from home may also promote certain aspects of health. Due to greater time flexibility, people may be less stressed by inflexible work schedules, thereby having the opportunity to align better their daily activities with their individual sleep/wake preference. Supporting this assumption, a study from Europe found that COVID-19-related restrictions, especially a lockdown, resulted in delayed bedtime, later rise times, increased sleep duration, and reduced social jetlag (shifts in sleep timing between workdays and free days) [5]—as also confirmed in a U.S. cohort [6]. Despite this evidence, it must be borne in mind that not all studies support observations of improved sleep during the early part of the pandemic [7,8,9]. For instance, an increasing number of psychiatric patients have been observed to complain about severe insomnia due to the pandemic and related restrictions [10]. Furthermore, according to a Greek study, sleep disturbances have been more intense for urban than rural residents [11]. Finally, in light of emerging evidence suggesting that COVID-19 can affect the brain [12], it is also possible that some changes in sleep that have been observed among people during the pandemic, may be causally resulted to effects of symptomatic and asymptomatic COVID-19.

Besides sleep, dietary choices may have also changed as a result of the COVID-19 pandemic and related restrictions. Supporting this assumption, a study from the United Arab Emirates involving 1012 subjects found more unhealthy dietary patterns during the pandemic. For instance, 46.1% of the participants consumed sweets and desserts at least once per day, and 37.1% reported consuming salty snacks (chips, crackers, and nuts) every day) [13]. In addition to diet quality, the timing of food intake may also be relevant to human health. Alterations in feeding time can uncouple the body clocks, leading to circadian misalignment, disruption in homeostasis, and disturbances in many metabolic functions [14,15,16]. Whether the shift of nighttime sleep to later hours during the pandemic has also shifted people’s eating time window toward later hours of the day is unknown. Later eating has been associated with various health outcomes, such as an increased risk of cancer, cardiometabolic complications, reduced efficacy of weight-loss interventions, lower insulin sensitivity, and adverse body composition [17,18,19], although such outcomes may partly be driven by a greater mismatch with fixed work schedules (akin to similar associations observed for those with greater social jetlag).

In the present study, we used data from 191 adults from Sweden, who at the time of the pandemic were surveyed about their sleep and meal timing habits both before and during the pandemic. Our hypothesis was that adults would exhibit a shift of habitual nighttime sleep to later hours on working days (due to, e.g., flexible work schedules) and, consequently, a reduction in social jetlag. We also hypothesized that delayed sleep schedules would occur concomitantly with a later onset and offset of the habitual meal timing windows.

## 2. Results

### 2.1. Cohort Characteristics

The anonymous survey was available online, in Swedish, between June and August 2020 (more details about the structure of the survey can be found in ref. [20]). It was administered through the Qualtrics platform (https://www.qualtrics.com accessed on 25 June 2020) and publicly accessible (until 10 August 2020). Of the questionnaire’s 869 respondents, 191 provided the full information required for the present analysis (for more details on exclusion criteria, see Table S1). Participation in the online survey was voluntarily, could be stopped at any time point by the respondent, and was not reimbursed. The final sample was on average 47.2 (13.1) years old, slightly overweight with BMI around 25.2 (4.7) (27.2% of respondents were overweight and 16.8% were obese), mainly female (77.5%), and the majority was either married or cohabiting (70.7%). A 13.1% share of the respondents stated having a definitely morning circadian preference, and 27.2% indicated a more morning than evening circadian preference. Additionally, 18.8% reported an intermediate chronotype. Finally, 24.1% stated that they had a more evening than morning circadian preference, and 16.8% stated having a definitely evening circadian preference.

### 2.2. Sleep before and Amid the Pandemic

Both before and during the pandemic, on working days, participants went earlier to bed, woke up earlier, exhibited an earlier sleep midpoint, and slept shorter compared with free days (*p* < 0.001 for all Wilcoxon signed-rank test comparisons; Table 1). Similarly, compared with free days, participants reported earlier time points for the first and last meal of the day (resulting in an earlier eating midpoint) and longer eating time windows on working days (*p* < 0.001 for all Wilcoxon signed-rank test comparisons). Again, these differences were observed both before and during the pandemic (Table 1).

**Figure 1:**
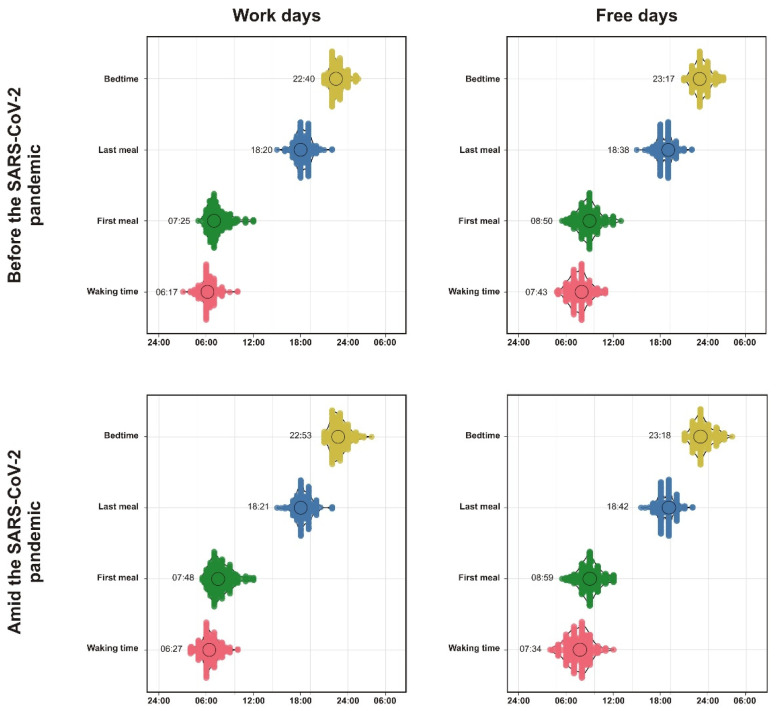
Meal and sleep timing variables before and amid the COVID-19 pandemic, split by work and free days. Mean value (in military time) is shown next to each figure element; y-axis shows frequency over clock time.

As summarized in Table 1 and Figure 1, on workdays but not free days, respondents went to bed significantly later during the pandemic compared to the pre-pandemic period (~13 min on average). Although the waking time was delayed by approximately 10 min on working days during the pandemic, following adjustment for multiple comparisons, the comparison with pre-pandemic waking time did not reach significance. We nonetheless found that the sleep midpoint on workdays was on average approximately 11 min later. No difference in sleep midpoint between pre-pandemic and pandemic on free days was found. Indicating that sleep times between work and free days were better aligned when compared with the pre-pandemic period, social jetlag significantly decreased during the pandemic (−17 min on average), but no difference in time in bed was observed.

### 2.3. Meal Timing before and Amid the Pandemic

Respondents scheduled their first meal about 23 min later on workdays and 9 min later on free days during the pandemic compared to the pre-pandemic period (Table 1 and Figure 1). In contrast, the timing of the last meal did not differ significantly between the pandemic vs. pre-pandemic period. Mainly driven by the delayed onset of the first meal, the eating midpoint was about 12 min later on workdays and 6 min later on free days during the pandemic compared with before the pandemic. We also observed that the length of the eating time window was significantly shorter on workdays during the pandemic (−21 min). Finally, eating jet lag did not differ between the pandemic and pre-pandemic periods.

Before and during the pandemic, significant correlations were observed between the sleep and eating midpoints, both on working and free days (Table 2), supporting the notion that meal timing is associated with sleep timing.

## 3. Discussion

In the present study, we found a relatively small but significant effect of the COVID-19 pandemic on sleep schedules. Compared to before, people went about 13 min later to bed during the pandemic. Reflecting how meal timing may be inherently tied to the sleep/wake cycle, we observed that a later bedtime and sleep midpoint during the COVID-19 pandemic were accompanied by a later scheduled first meal on workdays and free days. Notably, in contrast, the timing of the last meal amid the pandemic did not differ compared to before the pandemic. As respondents’ main change in eating patterns was a later start to their first meal of the day (meal onset), their eating time window was shorter on working days during the pandemic, and the evening fasting period was prolonged on working days. A shorter daily eating time window has been linked to improved metabolic health among obese and overweight subjects [21]. Longer duration of pre-sleep fasting may reduce the risk of gastroesophageal reflux disease [22]. Finally, delaying the first meal of the day prolongs the overnight fast (i.e., the time between the last meal of the preceding day and the first meal of the current day), which has been associated with a reduced likelihood of being overweight or obese [23]. Some of these effects may occur through modulation of the diurnal cortisol profile and meal-induced thermogenesis [24,25].

There were limitations to this study. There could have been bias in the range of responses, as our survey was distributed online without the ability to ensure responses covering a broad range of demographics. Our data were, furthermore, based on participant recall, which is also subject to bias. Many subjects who completed the survey were excluded for missing data, which may be explained by the lack of financial reimbursement for survey participation. Additionally, we excluded respondents with irregular working hours (e.g., night shift workers). This loss of data might have introduced selection bias. Residual confounding due to factors such as seasonality should also be taken into consideration when interpreting our findings.

Another important limitation was that we did not examine whether dietary choices differed between, before, and during the pandemic. A recent survey study from Italy revealed that respondents exhibited less healthy dietary choices during the pandemic than before, including but not limited to increased intake of simple sugar and sweets and decreased intake of legumes [26]. Thus, it is unclear whether possible health benefits related to a shorter eating time window and longer overnight fast, as observed in our study, might be offset by poor dietary choices during the pandemic.

Although we found that bedtime and sleep midpoint were delayed, sleep duration remained unchanged, which contrasts with previous reports of increased sleep duration during the COVID-19 pandemic [5,6]. In this context, it is important to bear in mind that in Sweden, sports events and large gatherings were banned, but business, cafes, and shops remained open for people during the period of our data collection in 2020. Looser restrictions and a significant number of daily COVID-19-related deaths may have contributed to poorer mental health and wellbeing in Sweden. As suggested by a Swedish survey study, 45.6% reported symptoms reflecting significant problems in one or more areas of their mental health, including depression, anxiety, or insomnia [27]. These adverse effects on mental health may have weakened possible positive effects of work schedule flexibility due to work at home recommendations on sleep duration.

## 4. Materials and Methods

### 4.1. Survey Procedure

With the primary aim to investigate the impact of the SARS-CoV-2 pandemic on sleep and circadian rhythms, an anonymous online survey study was performed across 14 countries/areas during 2020 (International COVID-19 Sleep Study, ref. [20]). Respondents were asked about the exact going to bed and waking up times on both work and free days before and during the pandemic. Cronbach’s alpha indicated acceptable internal consistency for questions on bedtime and waking time on working and free days (alpha = 0.897). Thus, we could calculate the time in bed and social jetlag (the difference in sleep midpoint between work and free days, ref. [28]). In Sweden only, respondents were also asked about meal timing. Specifically, the participants indicated the exact clock time of the first and last main meal on work and free days separately, both before and during the pandemic (Crohnbach’s alpha = 0.818). We then estimated the following (pre)pandemic meal timing characteristics: time of the first and last meal, the midpoint of the eating time window, eating jet lag (the difference in eating midpoint between work and free days), and length of eating time window. Information about the existence of the anonymous survey was circulated on TV, Internet, and social media platforms. The survey was presented as questionnaire aimed at investigating sleep and associated lifestyle habits during the COVID-19 pandemic. No information was provided about this paper’s expected outcome. Between 25 June 2020, and 10 August 2020, participants were able to respond to the questionnaire, which was presented in Swedish, via the online platform Qualtrics. Following exclusions (specified in Table S1), complete survey data of 191 anonymous respondents were available for analysis.

### 4.2. Statistical Analysis

Statistical analyses were performed using SPSS version 24 (SPSS Inc., Chicago, IL, USA). Due to the skewness of the data (confirmed by significant Shapiro-Wilk tests), we used the non-parametric Wilcoxon signed-rank test to examine whether sleep and meal timing variables differed within subjects between the pre-pandemic and pandemic periods, as well as between work and free days. Given the moderate sample size of the present study, our main analysis was restricted to the pandemic’s effects on meal and sleep timing parameters in the full cohort. Exploratory analyses, stratified by age, sex, and BMI—all known to affect circadian rhythms [29]—can be found in Tables S2–S4. Overall, a *p* value < 0.05 was considered significant. Where appropriate, Bonferroni correction was applied when testing multiple pairwise comparisons. If not otherwise stated, data are shown as mean (SD).

## 5. Conclusions

Our study suggests that later sleep times, as seen during the COVID-19 pandemic, prolong daily meal onset latency. The observation that the timing of the last meal remained unaffected by later sleep times could suggest that other variables not measured herein, such as the habit of having a regular dinner with the spouse or family, may play a more prominent role in determining when the last meal is consumed. Our findings must be confirmed by data collected in other countries and in larger populations that allow for more detailed analyses of possible confounding factors.

## Figures and Tables

**Table 1:** Sleep and meal timing before and amid the COVID-19 pandemic, on work and free days. Social jetlag was calculated by subtracting the sleep midpoint of workdays from the free days’ sleep midpoint. Eating jetlag was calculated by subtracting the meal midpoint of workdays from the free days’ meal midpoint. Due to skewness of the data, comparisons between the time points were analyzed with the non-parametric Wilcoxon signed-rank test. A *p*-value smaller than 0.0028 was considered significant (shown in bold; *p* = 0.05 divided by 18 comparisons). Data are shown as mean ± standard deviation (SD). Abbreviations: hh:mm, military time; hr.min, hours + minutes.

Parameter	Pre−Pandemic	Pandemic	*Z* Value	*p* Value
	Mean (SD)	Mean (SD)		
Workdays				
Bedtime (hh:mm)	22:40 (00:55)	22:53 (01:05)	−3.910	<0.001
Waking time (hh:mm)	06:17 (00:56)	06:27 (01:08)	−2.532	0.011
Sleep midpoint (hh:mm)	02:29 (00:49)	02:40 (00:57)	−4.204	<0.001
Time in bed (hr.min)	7.38 (0.56)	7.34 (1.08)	−1.035	0.301
First meal (hh:mm)	07:25 (01:16)	07:48 (01:22)	−6.013	<0.001
Last meal (hh:mm)	18:20 (00:59)	18:21 (01:02)	−0.235	0.814
Eating midpoint (hh:mm)	12:52 (00:50)	13:04 (00:55)	−4.715	<0.001
Eating time window (hr.min)	10.55 (1:32)	10.34 (1:35)	−4.939	<0.001
Free days				
Bedtime (hh:mm)	23:17 (01:03)	23:18 (01:13)	−0.033	0.973
Waking time (hh:mm)	07:43 (1.27)	07:34 (01:23)	−2.008	0.045
Sleep midpoint (hh:mm)	03:30 (01:02)	03:26 (01:09)	−1.456	0.145
Time in bed (hr.min)	8.26 (1.05)	8.16 (1.14)	−1.987	0.047
First meal (hh:mm)	08:50 (01:22)	08:59 (01:17)	−3.239	0.001
Last meal (hh:mm)	18:38 (01:02)	18:42 (01:02)	−1.586	0.113
Eating midpoint (hh:mm)	13:44 (00:59)	13.50 (00:57)	−3.158	0.002
Eating time window (hr.min)	9.47 (1.23)	9.43 (1.23)	−1.719	0.086
Social jetlag (hr.min)	1.04 (0.49)	0.46 (0.47)	−6.020	<0.001
Eating jetlag (hr.min)	0.52 (0.53)	0.47 (0.49)	−0.111	0.912

**Table 2:** Spearman correlation between sleep and eating midpoints before and during the pandemic. The table reports Spearman rank-order correlation coefficients. ** *p* value smaller than 0.0125 (Bonferroni corrected). Abbreviations: N/A, not applicable.

			Pre-Pandemic		Pandemic	
			Eating Midpoint		Eating Midpoint	
			Workdays	Free Days	Workdays	Free Days
Pre-pandemic	Sleep midpoint	Workdays	0.540 **	N/A	N/A	N/A
		Free days	N/A	0.697 **	N/A	N/A
Pandemic	Sleep midpoint	Workdays	N/A	N/A	0.478 **	N/A
		Free days	N/A	N/A	N/A	0.618 **

## Data Availability

Upon request from C.B. or J.C., the data can be shared with researchers.

## Supplementary Materials

The following are available online at https://www.mdpi.com/article/10.3390/clockssleep3020015/s1, Table S1: Exclusions; Table S2: Sleep and meal timing before and amid the COVID-19 pandemic, split by age; Table S3: Sleep and meal timing before and amid the COVID-19 pandemic, split by gender; and Table S4: Sleep and meal timing before and amid the COVID-19 pandemic, split by BMI.
