# Supplementary material for: Meal and Sleep Timing before and during the COVID-19 Pandemic: A Cross-Sectional Anonymous Survey Study from Sweden
Source: Clocks Sleep. 2021 Apr 22;3(2):251–8. doi: 10.3390/clockssleep3020015 (PMC8167780; doi:10.3390/clockssleep3020015)
Supplement: Supplementary file 1 [file clockssleep-03-00015-s001.zip › clockssleep-1147803-SI.pdf]

Benedict et al., Online supplement

Table S1: Exclusions

**Survey parameter**	**No (% of all respondents)**
Number of respondents who participated in the survey	869 (100)
Did not answer the meal timing questions	-445 (51.2)
Suffered from diseases that can impact dietary choices (diabetes, cancer, depression, kidney disease, circadian rhythm disorder, myalgic encephalomyelitis, chronic pulmonary disease, stroke, cardiovascular disease)	-54 (6.2)
Reported regular shift work (outside regular working hours), which can mask the impact of the pandemic on sleep and meal timing	-44 (5.1)
Missing data on bedtime or waking time	-1 (0.1)
Reported bedtime or waking time deviated ≥3 SD from the population mean	-25 (2.9)
Reported first or last meal time deviated ≥3 SD from the population mean	-19 (2.2)
Missing data on civil status	-8 (0.9)
Sleep midpoint deviated ≥3 SD from the population mean	-7 (0.8)
Excluded because of pregnancy	-7 (0.8)
Respondent did not reside in Sweden	-6 (0.7)
BMI deviated ≥3 SD from the population mean	-3 (0.3)
Time of the first meal time was earlier than waking time	-59 (6.8)
**Final cohort**	**191 (22.0%)**

Table S2: Sleep and meal timing before and amid the COVID-19 pandemic, split by age. The median age (47 years) was used to divide subjects into young and old adults. Subjects of the old age group (n=98; ≥median age) had the following characteristics (mean, SD or %group): age, 57.4 (8.7); females/males, 75.3%/24.7%; and BMI, 26.0 (4.4). Subjects of the young age group (n=93; 79.6% females) were on average 36.5 (7.1) years old and had a mean BMI of 24.4 (4.8) kg/m^2. Due to skewness of the data, comparisons between the time points were analyzed with the non-parametric Wilcoxon signed-rank test. A P-value smaller than 0.0013 was considered significant (Bonferroni corrected, shown in bold). Social jetlag was calculated by subtracting the sleep midpoint of work days from the free days' sleep midpoint. Eating jetlag was calculated by subtracting the meal midpoint of work days from the free days' meal midpoint.

**Age group**	**Parameter**	**Before**<br>Mean (SD)	**Amid**<br>Mean (SD)	***P*-value**
**Young**	**Work days**			
	Bedtime (hh:mm)	22:37 (00:55)	22:55 (01:09)	**0.001**
	Waking time (hh:mm)	06:21 (00:46)	06:33 (01:08)	0.018
	Sleep midpoint (hh:mm)	02:29 (00:45)	02:44 (01:00)	**<0.001**
	Time in bed (hr.min)	7.43 (0.43)	7.38 (1.07)	0.317
	First meal (hh:mm)	07:30 (01:20)	07:56 (01:24)	**<0.001**
	Last meal (hh:mm)	18:15 (00:57)	18:18 (01:04)	0.891
	Eating midpoint (hh:mm)	12:52 (00:49)	13:07 (00:58)	**<0.001**
	Eating time window (hr.min)	10.46 (1.37)	10.21 (1.36)	**<0.001**
	**Free days**			
	Bedtime (hh:mm)	23:21 (01:07)	23:23 (01:15)	0.622
	Waking time (hh:mm)	07:57 (01:26)	07:46 (01:33)	0.063
	Sleep midpoint (hh:mm)	03:39 (01:09)	03:34 (01:14)	0.302
	Time in bed (hr.min)	8.36 (1.11)	8.23 (1.21)	0.063
	First meal (hh:mm)	09:03 (01:35)	09:13 (01:27)	0.021
	Last meal (hh:mm)	18:36 (01:04)	18:40 (01:05)	0.493
	Eating midpoint (hh:mm)	13:50 (01:09)	13:56 (01:03)	0.041
	Eating time window (hr.min)	9.33 (1.27)	9.27 (1.26)	0.104
	**Social jetlag** (hr.min)	1.12 (0.50)	0.50 (0.49)	**<0.001**
	**Eating jetlag** (hr.min)	0.57 (0.57)	0.50 (0.51)	0.531
**Old**	**Work days**			
	Bedtime (hh:mm)	22:42 (00:56)	22:51 (01:01)	0.044
	Waking time (hh:mm)	06:15 (01:04)	06:21 (01:08)	0.178
	Sleep midpoint (hh:mm)	02:28 (00:52)	02:36 (00:55)	0.038
	Time in bed (hr.min)	7.34 (1.01)	7.30 (1.09)	0.622
	First meal (hh:mm)	07:21 (01:12)	07:40 (01:18)	**<0.001**

Last meal (hh:mm)	18:24 (01:00)	18:25 (01:00)	0.807
Eating midpoint (hh:mm)	12:52 (00:50)	13:02 (00:58)	**<0.001**
Eating time window (hr.min)	11.03 (1.27)	10.45 (01:33)	**<0.001**
**Free days**			
Bedtime (hh:mm)	23:14 (00:59)	23:14 (01:11)	0.765
Waking time (hh:mm)	07:30 (01:03)	07:22 (01:12)	0.311
Sleep midpoint (hh:mm)	03:22 (00:54)	03:18 (01:03)	0.283
Time in bed (hr.min)	8.16 (00:58)	8.09 (1.08)	0.371
First meal (hh:mm)	08:39 (01:05)	08:46 (01:06)	0.023
Last meal (hh:mm)	18:39 (01:00)	18:43 (01:00)	0.119
Eating midpoint (hh:mm)	13:39 (00:49)	13:45 (00:50)	0.013
Eating time window (hr.min)	10.01 (01.17)	9.57 (1.18)	0.531
**Social jetlag** (hr.min)	0.55 (0.46)	0.42 (0.45)	**<0.001**
**Eating jetlag** (hr.min)	0.47 (0.48)	0.44 (0.47)	0.601

Table S3: Sleep and meal timing before and amid the COVID-19 pandemic, split by gender. Females (n=148) had the following characteristics (mean, SD or %group): age, 47.8 (13.5); and BMI, 25.0 (4.7). Male subjects of the young age group (n=43) were on average 45.2 (11.8) years old and had a mean BMI of 25.9 (4.5) kg/m^2. Due to skewness of the data, comparisons between the time points were analyzed with the non-parametric Wilcoxon signed-rank test. A P-value smaller than 0.0013 was considered significant (Bonferroni corrected, shown in bold). Social jetlag was calculated by subtracting the sleep midpoint of work days from the free days' sleep midpoint. Eating jetlag was calculated by subtracting the meal midpoint of work days from the free days' meal midpoint.

**Gender**	**Parameter**	**Before**<br>Mean (SD)	**Amid**<br>Mean (SD)	***P*-value**
**Females**	**Work days**			
	Bedtime (hh:mm)	22:36 (00:57)	22:48 (01:06)	**0.001**
	Waking time (hh:mm)	06:17 (00:57)	06:26 (01:09)	0.030
	Sleep midpoint (hh:mm)	02:26 (00:50)	02:37 (00:59)	**<0.001**
	Time in bed (hr.min)	7.41 (0.55)	7.38 (1.06)	0.540
	First meal (hh:mm)	07:28 (01:17)	07:53 (01:23)	**<0.001**
	Last meal (hh:mm)	18:21 (00:56)	18:23 (00:57)	0.373
	Eating midpoint (hh:mm)	12:55 (00:50)	13:08 (00:54)	**<0.001**
	Eating time window (hr.min)	10.53 (1.31)	10.29 (1.33)	**<0.001**
	**Free days**			
	Bedtime (hh:mm)	23:10 (01:02)	23:09 (01:11)	0.816
	Waking time (hh:mm)	07:41 (01:18)	07:31 (01:27)	0.091
	Sleep midpoint (hh:mm)	03:25 (01:03)	03:20 (01:11)	0.134
	Time in bed (hr.min)	8.30 (1.05)	8.22 (1.14)	0.142
	First meal (hh:mm)	08:50 (01:22)	08:59 (01:14)	0.013
	Last meal (hh:mm)	18:38(01:01)	18:41 (00:58)	0.325
	Eating midpoint (hh:mm)	13:44 (00:59)	13:50 (00:53)	0.035
	Eating time window (hr.min)	9.48 (1.24)	9.43 (1.21)	0.145
	**Social jetlag** (hr.min)	1.00 (0.47)	0.43 (0.46)	**<0.001**
	**Eating jetlag** (hr.min)	0.50 (0.51)	0.43 (0.46)	0.636
**Males**	**Work days**			
	Bedtime (hh:mm)	22:53 (00:45)	23:10 (00:59)	0.030
	Waking time (hh:mm)	06:20 (00:53)	06:29 (01:07)	0.078
	Sleep midpoint (hh:mm)	02:36 (00:40)	02:49 (00:52)	0.005
	Time in bed (hr.min)	7.27 (0.56)	7.19 (1.12)	0.363
	First meal (hh:mm)	07:15 (01:13)	07:28 (01:13)	0.036
	Last meal (hh:mm)	18:14 (01:09)	18:15 (01:18)	0.577
	Eating midpoint (hh:mm)	12:44 (00:51)	12:52 (00:57)	0.052

Eating time window (hr.min)	10.59 (1.39)	10.47 (1.40)	0.089
**Free days**			
Bedtime (hh:mm)	23:42 (01:01)	23:48 (01:12)	0.454
Waking time (hh:mm)	07:51 (01:10)	07:43 (01:09)	0.306
Sleep midpoint (hh:mm)	03:46 (00:58)	03:45 (01:00)	0.796
Time in bed (hr.min)	8.08 (1.01)	7.55 (1.15)	0.177
First meal (hh:mm)	08:51 (01:23)	09:00 (01:30)	0.008
Last meal (hh:mm)	18:36 (01:04)	18:44 (01:14)	0.206
Eating midpoint (hh:mm)	13:44 (01:02)	13:52 (01:09)	0.012
Eating time window (hr.min)	9.45 (1.21)	9.44 (1.30)	0.259
**Social jetlag** (hr.min)	1.13 (0.53)	0.56 (0.48)	**<0.001**
**Eating jetlag** (hr.min)	1.00 (0.58)	1.01 (0.57)	0.484

Table S4: Sleep and meal timing before and amid the COVID-19 pandemic, split by BMI. Participants with either overweight or obesity (i.e., ≥25 kg/m^2; n=84) had the following characteristics (mean, SD or %group): age, 50.0 (12.7); females/males, 76.2%/23.8%; and BMI, 29.5 (3.6). Subjects with a BMI smaller than 25 kg/m^2 (n=107; 78.5 % females) were on average 45.0 (13.1) years old and had a mean BMI of 21.9 (1.9) kg/m^2. Due to skewness of the data, comparisons between the time points were analyzed with the non-parametric Wilcoxon signed-rank test. A P-value smaller than 0.0013 was considered significant (Bonferroni corrected, shown in bold). Social jetlag was calculated by subtracting the sleep midpoint of work days from the free days' sleep midpoint. Eating jetlag was calculated by subtracting the meal midpoint of work days from the free days' meal midpoint.

**BMI**	**Parameter**	**Before** Mean (SD)	**Amid** Mean (SD)	***P*-value**
**<25 kg/m^2**	**Work days**			
	Bedtime (hh:mm)	22:40 (00:53)	22:50 (01:02)	0.004
	Waking time (hh:mm)	06:13 (00:48)	06:31 (01:02)	**<0.001**
	Sleep midpoint (hh:mm)	02:26 (00:43)	02:40 (00:52)	**<0.001**
	Time in bed (hr.min)	7.33 (0.52)	7.41 (1.05)	0.190
	First meal (hh:mm)	07:22 (01:19)	07:45 (01:23)	**<0.001**
	Last meal (hh:mm)	18:20 (00:58)	18:23 (01:01)	0.498
	Eating midpoint (hh:mm)	12:51 (00:50)	13:04 (00:56)	**<0.001**
	Eating time window (hr.min)	10.58 (1.36)	10.38 (1.33)	**<0.001**
	**Free days**			
	Bedtime (hh:mm)	23:14 (00:56)	23:16 (01:10)	0.745
	Waking time (hh:mm)	07:46 (01:13)	07:39 (01:19)	0.310
	Sleep midpoint (hh:mm)	03:30 (00:56)	03:28 (01:04)	0.565
	Time in bed (hr.min)	8.32 (1.05)	8.23 (1.15)	0.175
	First meal (hh:mm)	08:48 (01:24)	09:01 (01:21)	**<0.001**
	Last meal (hh:mm)	18:34 (01:03)	18:38 (01:06)	0.300
	Eating midpoint (hh:mm)	13:41 (01:00)	13:50 (00:59)	0.004
	Eating time window (hr.min)	9.46 (1.27)	9.37 (1.27)	0.041
	**Social jetlag** (hr.min)	1.06 (0.47)	0.48 (0.40)	**<0.001**
	**Eating jetlag** (hr.min)	0.50 (0.51)	0.47 (0.47)	0.880
**≥25 kg/m^2**	**Work days**			
	Bedtime (hh:mm)	22:40 (00:59)	22:57 (01:09)	0.008
	Waking time (hh:mm)	06:23 (01:05)	06:22 (01:16)	0.765
	Sleep midpoint (hh:mm)	02:32 (00:54)	02:39 (01:03)	0.034
	Time in bed (hr.min)	7.43 (1.00)	7.25 (1.11)	0.005

First meal (hh:mm)	07:29 (01:12)	07:51 (01:20)	**<0.001**
Last meal (hh:mm)	18:19 (01:01)	18:19 (01:04)	0.717
Eating midpoint (hh:mm)	12:54 (00:50)	13:05 (00:54)	0.018
Eating time window (hr.min)	10.50 (1.28)	10.28 (1.37)	**0.001**
**Free days**			
Bedtime (hh:mm)	23:21 (01:11)	23:20 (01:17)	0.690
Waking time (hh:mm)	07:39 (01:21)	07:26 (01:29)	0.074
Sleep midpoint (hh:mm)	03:30 (01:09)	03:23 (01:15)	0.112
Time in bed (hr.min)	8.17 (1.03)	8.07 (1.14)	0.163
First meal (hh:mm)	08:54 (01:20)	08:56 (01:14)	0.309
Last meal (hh:mm)	18:42 (01:01)	18:46 (00:58)	0.189
Eating midpoint (hh:mm)	13:48 (00:59)	13:51 (00:53)	0.142
Eating time window (hr.min)	9.49 (1.18)	9.50 (1.18)	0.971
**Social jetlag** (hr.min)	1.00 (0.51)	0.44 (0.55)	**<0.001**
**Eating jetlag** (hr.min)	0.54 (0.56)	0.47 (0.51)	0.679
